# Tomato ubiquitinome in response to ‘*Candidatus* Liberibacter solanacearum’ haplotypes A and B

**DOI:** 10.1007/s44297-026-00075-6

**Published:** 2026-05-11

**Authors:** Julien G. Levy, Jiaxing Liu, Junepyo Oh, Azucena Mendoza Herrera, Cecilia Tamborindeguy

**Affiliations:** 1 Department of Horticultural Sciences, Texas A&M University, College Station, TX 77843 USA; 2 Department of Entomology, Texas A&M University, College Station, TX 77843 USA

**Keywords:** Bacteria, *Candidatus* Liberibacter asiaticus, Effector, HPE1, Huanglongbing, Ubiquitylome, Zebra chip

## Abstract

‘*Candidatus* Liberibacter solanacearum’ (Lso) is a devastating bacterial pathogen of crops transmitted by psyllids. Previously, a link between changes in ubiquitination and the progression of diseases caused by Lso was established. Among the Lso haplotypes, LsoA and LsoB infect solanaceous crops. LsoA induces chlorosis and stunting in tomato, while LsoB infection leads to more severe symptoms, including plant death. To better characterize the molecular basis of the differences in the diseases caused by these haplotypes, we cataloged the ubiquitinome of tomato leaves four weeks after LsoA and LsoB-infection and following infestation with Lso-free psyllids. Our analysis revealed haplotype-specific changes in the plant ubiquitinome. Gene Ontology (GO) terms related to glucose metabolism and proteasome-mediated ubiquitin-dependent protein catabolism were enriched among the differentially ubiquitinated proteins between LsoA-infected and Lso-free infested plants. Additionally, a KEGG pathway analysis determined an overrepresentation of proteins involved in carbon metabolism and fixation via the Calvin cycle. In contrast, the comparison of LsoB-infected and Lso-free infested plants revealed a broader range of enriched GO terms and KEGG pathways, including those associated with carbohydrate and energy metabolism, proteasome activity, and oxidative stress. These results contribute to our understanding of the development of Liberibacter-related diseases and reveal the importance of ubiquitin-related pathways in Lso infection. They also contribute to the expanding inventory of ubiquitinated proteins.

**Supplementary Information:**

The online version contains supplementary material available at 10.1007/s44297-026-00075-6.

## Introduction

Liberibacter pathogens cause devastating diseases in crops worldwide [1]. To date, the mechanisms deployed by these pathogens to evade plant immunity and the host responses leading to symptom development remain poorly understood [2]. Depending on the pathosystem, liberibacters range from highly virulent, such as ‘*Candidatus* Liberibacter solanacearum’ (Lso) in potatoes, to commensal, such as ‘*Ca.* L. europaeus’ in pears [3]. Comparing the diseases associated with different Liberibacter species or strains can help elucidate the molecular events leading to disease development. For instance, several Lso haplotypes have been identified; among them, A and B affect members of the Solanaceae in the Americas and/or New Zealand [4–6]. Our laboratory has characterized the diseases associated with LsoA and LsoB in different hosts and found that LsoB is more virulent than LsoA in tomato and tobacco plants [7, 8]. In tomato plants, the symptoms associated with these two haplotypes appear after four weeks post-infection and include leaf deformation and chlorosis. As the disease develops, LsoA-infected tomato plants develop small leaves on the apical meristem, new growth from axillary meristems, and stunted growth; however, LsoB-infected plants develop more severe symptoms, including necrosis and plant death, 8 weeks after infection [7].

To investigate the mechanisms deployed by Lso to infect plants, several Lso effector proteins have been characterized [8–11]. Effectors are secreted proteins that can modulate plant immune responses. The first characterized Lso effector, HPE1, interacts with RADIATION SENSITIVE 23 (RAD23) proteins [12, 13]. RAD23 proteins act as "ubiquitin receptors" and function as shuttles to deliver ubiquitinated proteins to the proteasome for degradation. This function is enabled by their unique domain structure, which includes a ubiquitin-like (UbL) domain for proteasome interaction and ubiquitin-associated (UBA) domains for binding to ubiquitin chains. Yeast two-hybrid library screening revealed that HPE1 interacts with the UbL domain of tomato RAD23e. Furthermore, this effector also interacts with tomato RAD23c and Rad23d but not RAD23a. HPE1 expression in *Nicotiana benthamiana* leaves treated with the proteasome inhibitor MG132 resulted in the accumulation of ubiquitinated proteins. A similar accumulation was observed in LsoB-infected tomato leaves [12]. Therefore, through its interaction with RAD23, HPE1 could affect the degradation of proteins by the proteasome.

The ubiquitin–proteasome system (UPS), a crucial system for pathogen detection and defense deployment, is often targeted by pathogen effectors [14]. For example, phloem-restricted bacterial phytoplasmas secrete SAP54 and other phyllogen effectors (named after their specific phenotype) that interact with RAD23’s UBA domains, hijacking the ubiquitin-mediated degradation pathway and targeting specific plant transcription factors for degradation [15, 16]. While the functional consequences of HPE1 interaction with RAD23 proteins need to be elucidated, the overexpression of HPE1 accelerates the onset of symptoms in laboratory conditions. This evidence suggests a direct involvement of the plant ubiquitinome during Lso infection.

Ubiquitination is a post-translational attachment of ubiquitin, a 76-amino acid conserved protein, to substrates at a selected site. The best characterized function of ubiquitination is targeting proteins to the 26S proteasome complex for degradation. However, ubiquitination can also affect protein–protein interactions, as well as protein localization and stability. Therefore, manipulation or changes in the ubiquitination pathway play a key role in disease development [14, 17, 18]. The interaction between pathogen proteins and UPS components can be part of the host immune response to the pathogen attack or part of the pathogen infection strategy.

To shed light on the role of ubiquitination during Lso infection, we used ubiquitin remnant profiling to compare the ubiquitinated proteins of tomato leaves four weeks after LsoA and LsoB infection. Lso is characterized by a heterogeneous distribution in plants, i.e., LsoA and LsoB haplotypes differ in their distribution within the plant and in titer across tissues [7]. Despite these differences in localization and abundance, Lso-induced disease is systemic. At four weeks after infection, the top leaves of infected tomato plants have similar LsoA and LsoB titers [7]; later, as the disease progresses, LsoA and LsoB titers evolve distinctly. Therefore, here, to compare materials with similar Lso levels before differential symptoms develop, we evaluated the ubiquitinome of top leaves at four weeks after infection. This timepoint represents a relatively early time in the infection: symptoms such as stunting and leaf deformities are starting to develop, but differences between LsoA and LsoB infection are not yet discernible [7]. Our objective was to identify changes in the ubiquitination of proteins involved in immune responses and metabolism to shed light on the mechanisms leading to plant disease and tolerance to LsoA infection. This study revealed haplotype-specific changes in tomato ubiquitinome proteins and identified potential key proteins involved in the response to Lso infection. Furthermore, this study contributes to the growing field of ubiquitination dynamics in diseases.

## Results

### Infection with LsoA and LsoB results in distinct ubiquitinomes

The ubiquitinomes of LsoA- (LsoA) and LsoB-infected (LsoB) and uninfected (Lso-free) tomato leaf samples were obtained (n = 4). A total of 48,605 precursors were identified; of those, 4,070 were GlyGly ubiquitinated, corresponding to 941 ubiquitinated proteins.

**Fig. 1:**
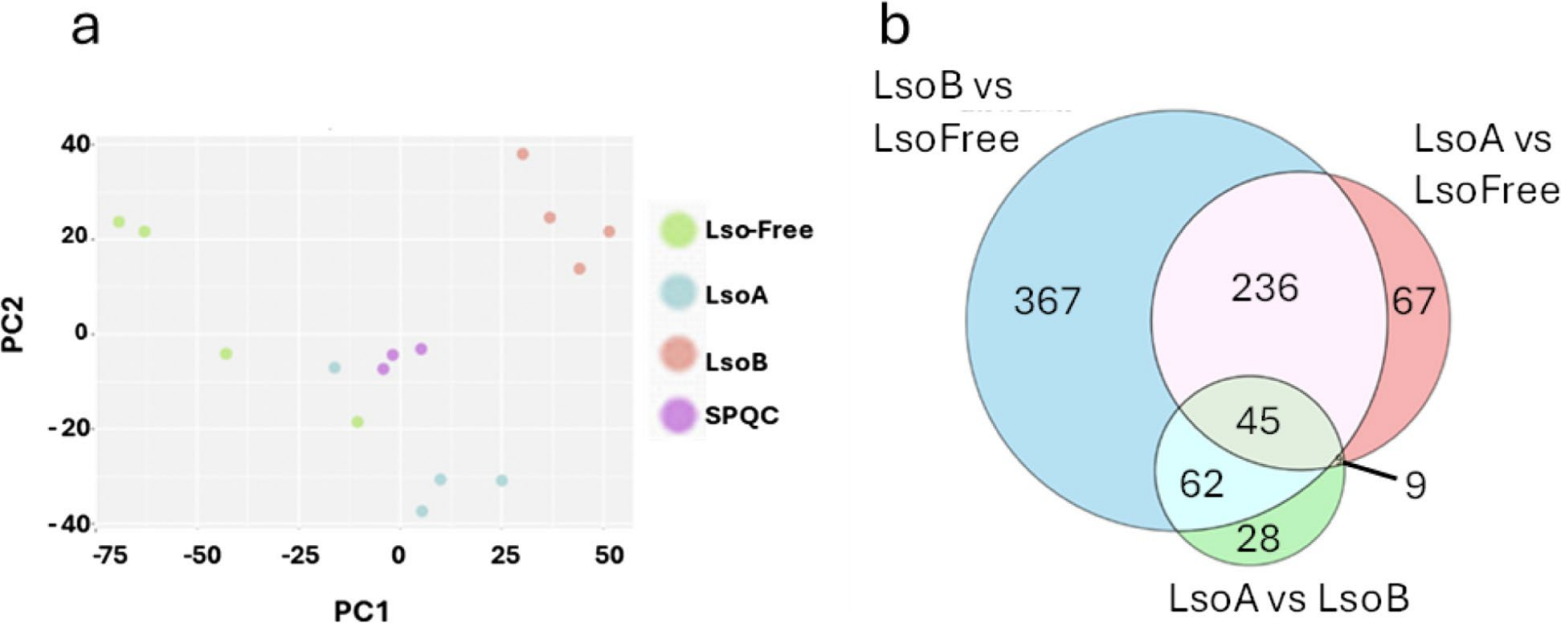
Ubiquitinome analysis. **a** PCA of –GlyGly peptide expression data (log2 Z-Scored) indicating a good separation of the three groups as a function of infection. It also showed the grouping of the SPQC control samples near the middle of the PCA. PC1 explains 32.80% of the variation and PC2 13.20%. **b** Venn diagram of differentially represented proteins in the ubiquitinomes of Lso-free, LsoA- and LsoB-infected tomato plants. The diagram was created with Venny 2.0 and modified with Microsoft Copilot

The PCA analysis of –GlyGly peptide expression data (log2 Z-Scored) indicated a good separation of the three groups as a function of the infection. The SPQC control samples were located near the middle of the PCA (Fig. 1a). These results indicate that LsoA and LsoB infection cause specific changes in the plant ubiquitinome.

There were 1190, 1234, and 1206 proteins ubiquitinated in each of the four LsoA, LsoB, and Lso-free biological replicates, represented by a total of 2900, 3065, and 2811 precursors, respectively. These numbers indicate an increase in protein ubiquitination following LsoB infection.

To identify the effect of LsoA- or LsoB- infection on the tomato ubiquitinome, three comparisons were conducted: Lso-free vs LsoA, Lso-free vs LsoB, and LsoA vs LsoB. Proteins with differential representation in the ubiquitinome were determined based on a p-adjusted value < 0.05 and signal detected in at least 75% of the replicates of one treatment.

A total of 357 accessions were identified as differential when comparing LsoA to Lso-free plants (170 were over-represented in the LsoA treatment) (Table S1), 710 accessions when comparing LsoB to Lso-free (354 of which were overrepresented in the LsoB treatment) (Table S2), and 144 when comparing LsoB to LsoA (61 were over-represented in the LsoA and 83 were over-represented in the LsoB) (Table S3) (Fig. 1b). These results support our previous findings that LsoB induces more changes in tomato gene expression than LsoA [19]. Furthermore, 281 accessions were common between the LsoA vs Lso-free and LsoB vs Lso-free comparisons (Fig. 1b, Table S4). These accessions can represent core responses to Lso infection and could include proteins involved in the common symptom development process and immune responses.

### Gene ontology and Kyoto encyclopedia of genes and genomes analyses of liberibacter-infected tomato ubiquitinomes

**Fig. 2:**
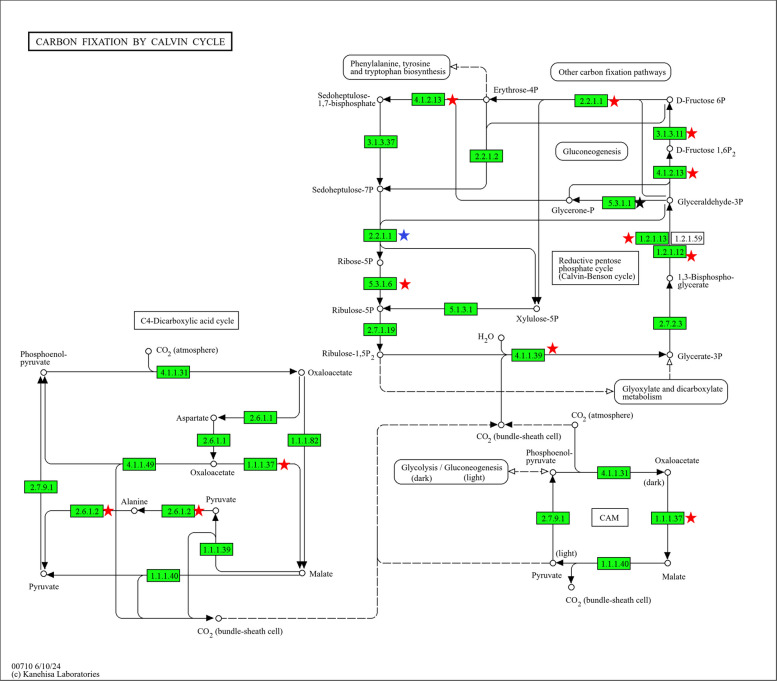
Proteins involved in Carbon fixation by Calvin cycle were differentially represented in the ubiquitinome of liberibacter-infected tomato plants. Red stars represent differential proteins identified in the LsoA vs Lso-free and LsoB vs Lso-free comparisons; the blue star identifies a protein identified as differential only in the LsoA vs Lso-free comparison; and the black star identifies a protein identified as differential only in the LsoB vs Lso-free comparison

To obtain a global view of the changes in the tomato ubiquitinome induced by LsoA and LsoB infection, we performed GO term and KEGG pathway enrichment analyses using the DAVID Functional Annotation Tool with the proteins identified as differentially enriched between the Lso-infected and uninfected samples (Table S5 and Fig. S1). In response to LsoA infection, there were two enriched biological process terms (glucose metabolic and proteasome-mediated ubiquitin-dependent protein catabolic processes), three enriched molecular function terms (GAPDH activity and ubiquitin and polyubiquitination binding), and three enriched cellular component terms (associated with cytoplasm, cytosol, and proteasome). There were also two enriched KEGG pathways: carbon fixation by the Calvin cycle and carbon metabolism (Fig. 2).

**Fig. 3:**
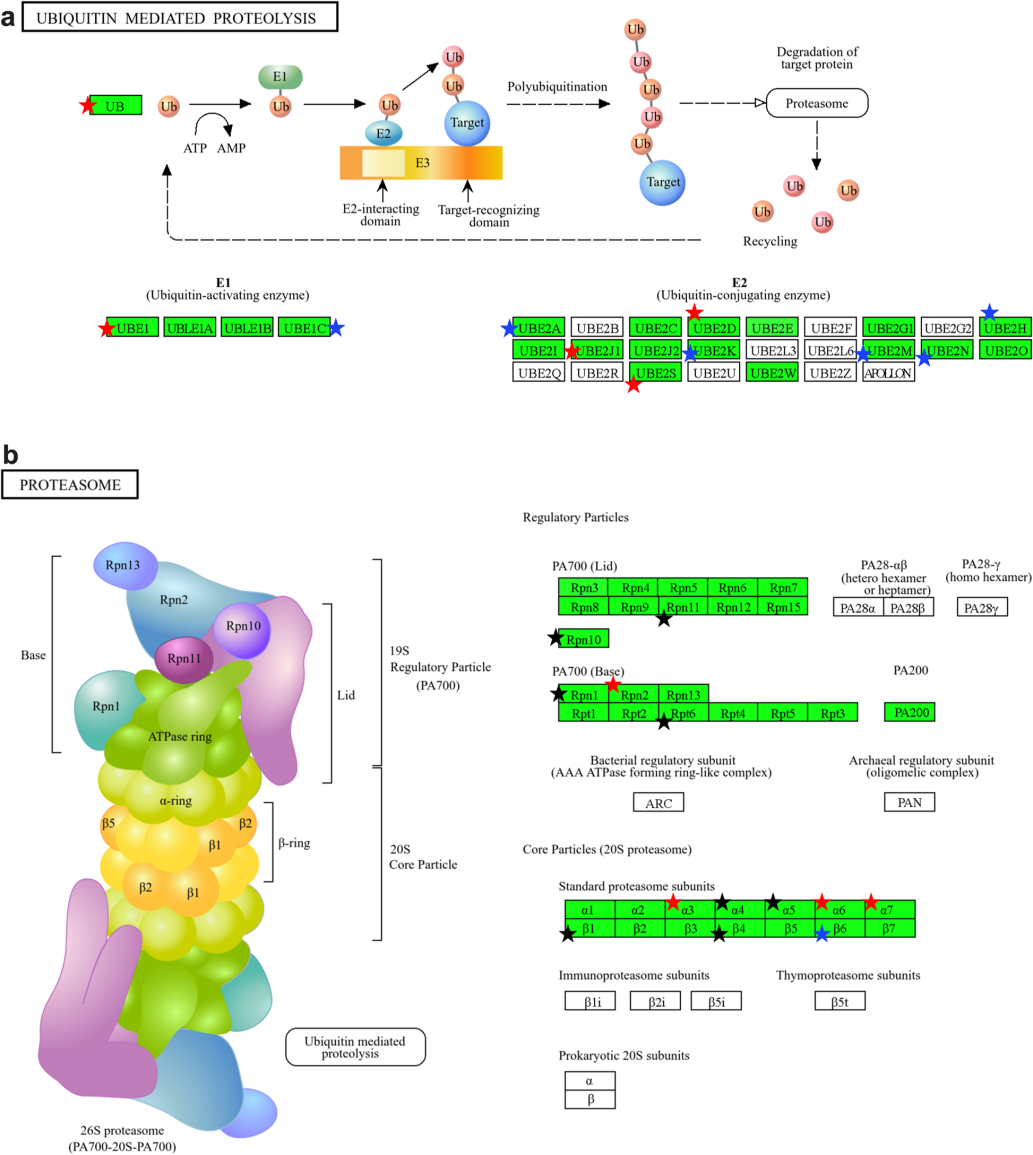
Proteins involved in Ubiquitin-mediated proteolysis (a, top) and Proteasome (b, bottom) were differentially represented in the ubiquitinome of liberibacter-infected tomato plants. Red stars represent differential proteins identified in the LsoA vs Lso-free and LsoB vs Lso-free comparisons. The blue stars identify proteins differential only in the LsoA vs Lso-free comparison, and the black star identify proteins differential only in the LsoB vs Lso-free comparison

In response to LsoB, there were more enriched terms: eleven biological process (related to sugar and energy metabolism, transport, proteasome processes, and translation and oxidative stress), sixteen molecular function terms (related to ubiquitination, translation, transport, and GAPDH activity, among others) and ten cellular compartment terms (related to cytoplasm, cytosol, proteasome, ribosome, chloroplast and nucleosome). There were also seven enriched KEGG pathways related to carbohydrate and energy metabolism, amino acid synthesis, endocytosis, and the proteasome (Figs. 2 and 3). A summary of the number of over- and underrepresented accessions in each comparison is provided in Table S6.

To assess “core changes” in the plant ubiquitinome related to Lso infection, we performed GO term and KEGG pathway enrichment analyses using the 236 proteins common to both comparisons. Two GO biological process terms (proteasome-mediated ubiquitin-dependent protein catabolic process and proton transmembrane transport) and three cellular compartment terms (cytoplasm, cytosol, proteasome core complex, alpha-subunit complex) were enriched. None of the Molecular Function terms were enriched. Similarly, only the KEGG pathway carbon fixation by Calvin cycle was enriched between the two comparisons. We also evaluated the 144 proteins that were differentially represented between LsoA and LsoB. Their analysis revealed significant enrichment in two molecular function terms (glyceraldehyde-3-phosphate dehydrogenase (NAD +) (phosphorylating) and water channel activities), two cellular compartment terms (cytoplasm and cytosol), and two KEGG pathways (carbon fixation by Calvin cycle and carbon metabolism). These results suggest that the diseases associated with these two haplotypes affect carbon assimilation by the plants and the activity of the proteasome; however, a key difference between these haplotypes is their effect on water transport.

Overall, 281 proteins were shared between the LsoA vs Lso-free and LsoB vs Lso-free comparisons; these included 236 differentially ubiquitinated proteins between the infected and Lso-free treatments and 45 differentially ubiquitinated proteins in the LsoA vs LsoB comparison (Fig. 1b). Only 18 proteins showed a fold change in the opposite direction when comparing LsoA vs Lso-free and LsoB vs Lso-free. While the tomato diseases associated with these haplotypes have very different outcomes, the changes induced at the ubiquitinome level early in the diseases are relatively similar. The differences in disease development seem to be driven by a few major differences.

### Evaluation of the ubiquitination and degradation of selected proteins

**Fig. 4:**
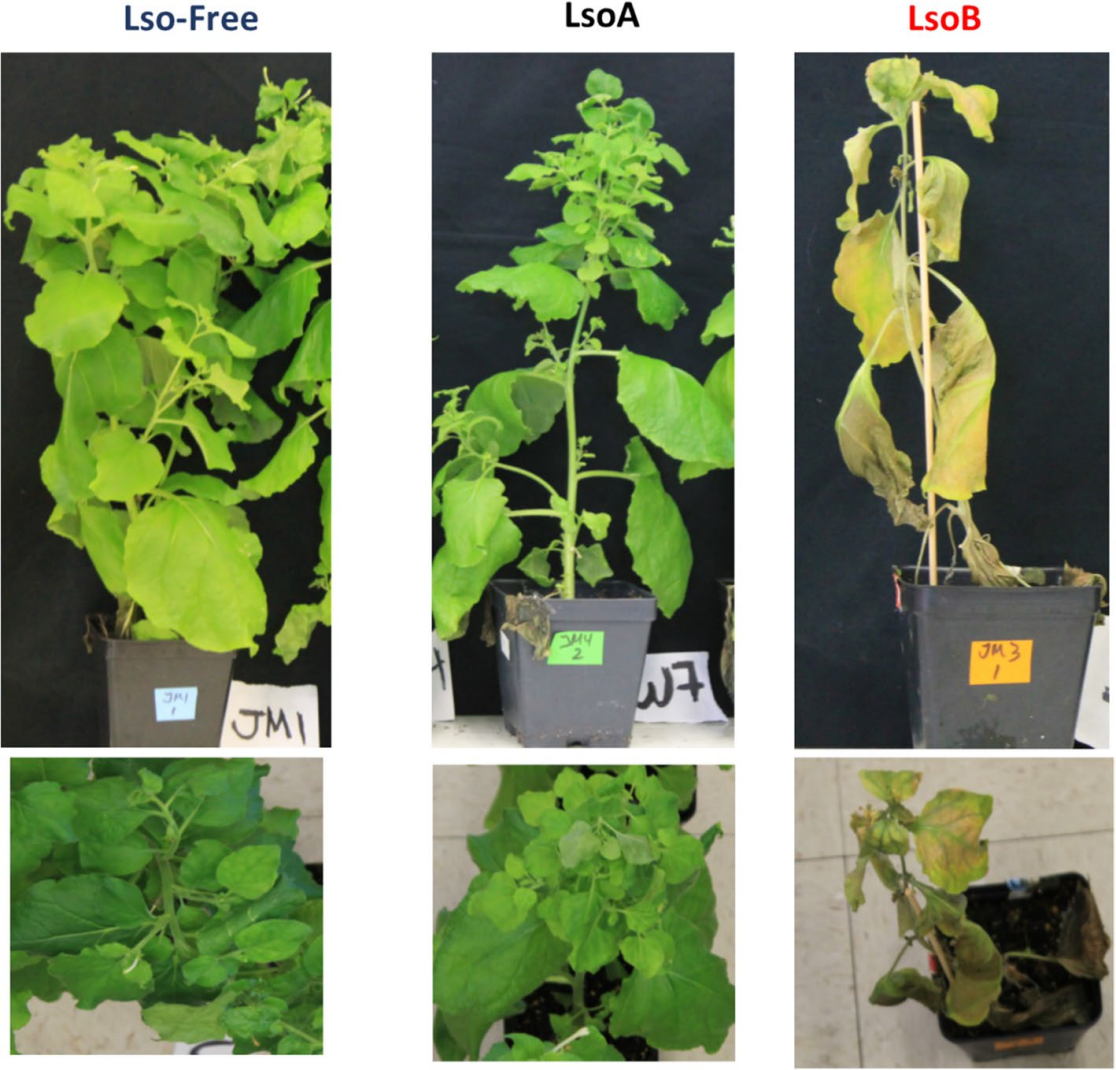
*Nicotiana benthamiana* 7 weeks after removing the psyllids for infection. The left panels show a control plant (infested with Lso-Free insects). The middle panels show a plant infected with LsoA: the plant is alive but less vigorous than the Lso-Free plant; of notice, small leaves in the new growth. The right panel shows a plant infected with LsoB: the infected plant is dead by the 7th week after infestation by LsoB insects. For size reference, the pots are 9 cm square pots

The potential ubiquitination and degradation by the proteasome of seven proteins was verified in Lso-free, LsoA- and LsoB-infected *N. benthamiana* plants. This assay was conducted to evaluate whether the differential representation of these proteins in the ubiquitinome datasets could be linked to their differential degradation by the proteasome, depending on the plant’s infectious status. *Nicotiana benthamiana* is a suitable model plant to investigate this question because LsoA and LsoB infection have similar outcomes in this host as in tomato (Fig. 4), but its leaves are easier to infiltrate and well-suited for transient protein expression.

**Fig. 5:**
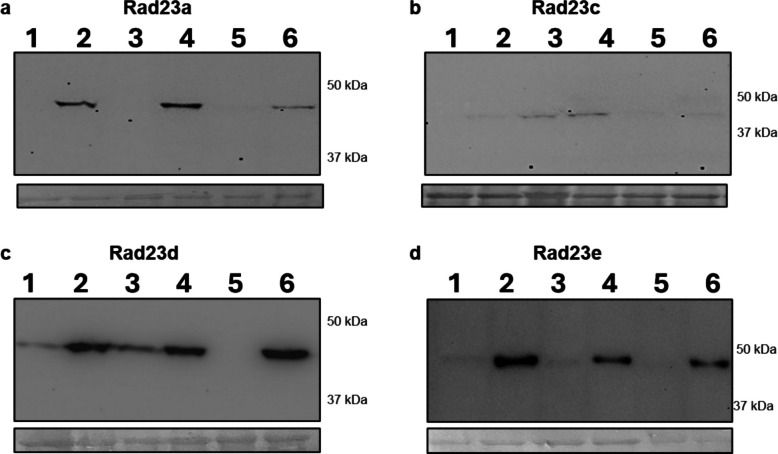
Detection of tomato RAD23 proteins overexpressed in LsoA-, LsoB-infected, and Lso-free *N. benthamiana* leaves. The leaves were infiltrated with *A. tumefaciens* strain GV3101 carrying the Rad23 genes subcloned into the pMDC32-HA binary vector fused with an HA tag and driven by 35SCaMV. At 60 h post-infiltration, the leaves were subjected to a second infiltration with either MG132 or DMSO and 12 h later samples were collected for Western blot detection using an anti-HA polyclonal antibody. **a** Rad23a; **b** Rad23c; **c** Rad23d; and **d** Rad23e, Lanes 1 and 2: Lso-Free; 3 and 4: LsoB; 5 and 6: LsoA. Lanes 1, 3 and 5 were treated with DMSO only, whereas 2, 4, and 6 were treated with MG132. For each protein, the top image is a photo of the Western blot, and the bottom is a ponceau staining

RAD23 proteins function as shuttle factors that transport ubiquitinated substrates to the proteasome, playing a crucial role in protein turnover and cellular stress responses. Four RAD23 proteins are encoded in the tomato genome: RAD23a, RAD23c, RAD23d, and RAD23e. RAD23 proteins have already been identified as potential targets during Lso infection, as RAD23c, RAD23d, and RAD23e interact with the effector Lso-HPE1 [12]. Here, RAD23d and RAD23e were identified among the differentially represented proteins in the ubiquitinome analysis (Tables S2 and S3). Specifically, two motifs corresponding to RAD23d had opposite representations in the LsoA vs Lso-free comparison, while only one of the motifs was underrepresented in LsoB compared to Lso-free. Additionally, RAD23e was overrepresented in LsoA and underrepresented in LsoB when compared to Lso-free. Therefore, we expressed the four proteins in *N. benthamiana* plants (Fig. 5). Irrespective of the plant’s infectious status, RAD23a and RAD23e could be detected only when the leaves were treated with MG132; therefore, these proteins appear to be degraded by the proteasome. On the other hand, RAD23d was detected in Lso-uninfected and LsoB-infected plants, but the intensity of the band increased when the inhibitor was used, suggesting some level of degradation by the proteasome. The difference in band intensity when comparing the signal from the MG132-treated and untreated samples suggests that a larger proportion of the protein was degraded in the Lso-uninfected leaves. However, in LsoA-infected plants, RAD23d was detected only when MG132 was used, meaning that this protein was degraded by the proteasome. Faint signals were obtained for RAD23c; the accumulation of this protein could not be evaluated.

**Fig. 6:**
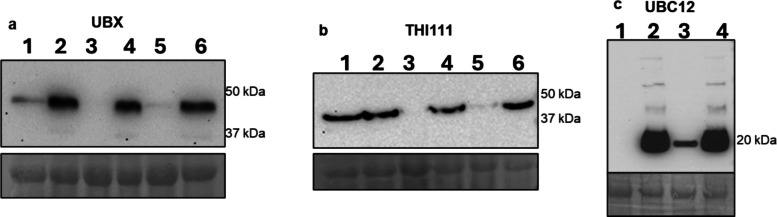
Detection of three tomato proteins overexpressed in LsoA-, LsoB-infected, and Lso-free *N. benthamiana* leaves. The leaves were infiltrated with *A. tumefaciens* strain GV3101 carrying the genes encoding for the proteins (**a**) UBX (Solyc07g049260.2), (**b**) TH1111 (Solyc10g005890.3.1), and (**c**) UBC12 (Solyc07g066080.2) subcloned into the pMDC32-HA binary vector fused with an HA tag and driven by 35SCaMV. At 60 h post-infiltration, the leaves were subjected to a second infiltration with either MG132 or DMSO and 12 h later samples were collected for Western blot detection using an anti-HA polyclonal antibody. Lanes 1 and 2: Lso-Free; 3 and 4: LsoB; 5 and 6: LsoA. Lanes 1, 3 and 5 were treated with DMSO only, whereas 2, 4, and 6 were treated with MG132. For each protein, the top image is a photo of the Western blot, and the bottom is a ponceau staining

We also evaluated the degradation of PLANT UBX DOMAIN-CONTAINING PROTEIN 4 and HOP-INTERACTING PROTEIN THI111 because of their potential involvement in proteasome degradation. Both proteins were over-represented in LsoB compared to Lso-free samples. In vivo expression of the HA-tagged candidates showed that these proteins were detected only in LsoB-infected plants when the proteasome inhibitor MG132 was added. In LsoA-infected plants, a faint band was observed in the absence of MG132, while in Lso-free samples, a band was observed (Fig. 6a and b). These results confirm that these proteins are degraded in LsoB-infected plants, which is in agreement with the ubiquitinome results.

NEDD8-CONJUGATING ENZYME UBC12 functions as an E2 ubiquitin-conjugating enzyme, facilitating ubiquitin transfer in various cellular processes, including stress responses and protein quality control. UBC12 was under-represented in LsoB samples compared to Lso-free samples. Our western blotting results confirmed this profile: UBC12 was detected only in the Lso-free samples when treated with MG132, while it was detected in LsoB-infected samples even without the addition of the proteasome inhibitor (Fig. 6c).

### Transcript expression of Rad23 is not altered by Lso infection

The expression of the Rad23 genes following Lso infection was evaluated in tomato plants four weeks after infestation. Lso infection did not affect the expression of any of the Rad23 transcripts (*p-value* > 0.05) (Fig. S2).

## Discussion

Protein ubiquitination plays a central role in maintaining cell homeostasis and is a key component of the cell defense strategy against infection. Plants recognize pathogens or their products and trigger pattern-triggered immunity (PTI) and/or effector-triggered immunity (ETI) [20, 21]. Protein ubiquitination is a dynamic process involved in both PTI and ETI signaling [22–24]. Recent advances in proteomics have enabled the high-throughput profiling of ubiquitinomes, offering comprehensive insights into the role of this post-translational modification in the organism's adaptation to various physiological conditions.

Previously, we characterized HPE1, an Lso effector that interacts with tomato RAD23 proteins, and we determined that LsoB-infected plants accumulate ubiquitinated proteins [12]. To further investigate the role of ubiquitination during Lso infection and symptom development, we cataloged the tomato ubiquitinome in response to two Lso haplotypes. The study revealed extensive haplotype-specific changes in protein ubiquitination. The overall ubiquitinome results aligned with previous transcriptome analyses, which determined haplotype-specific changes in the tomato transcriptome four weeks after Lso infection and more pronounced changes in response to LsoB [19].

In response to both haplotypes, proteins involved in carbohydrate and energy metabolism, including those in the Calvin cycle, were affected. Most of the precursor proteins in the Calvin cycle were under-represented in the infected samples. While chlorosis is not yet obvious four weeks after infection, LsoA- and LsoB-infected plants develop chlorosis as the disease progresses [7]. The underrepresentation of these proteins in the infected samples suggests that the molecular changes leading to this symptom occur earlier in the infection process. Furthermore, Lso is a phloem-restricted pathogen that disrupts sugar metabolism in infected plants [19, 25, 26]. Several over- and underrepresented proteins identified between the control and infected plants were related to glycolysis, sugar metabolism, and energy metabolism. These changes could be a consequence of the pathogen's energetic needs and be associated with changes in the plant source-sink relationship. These findings enhance the understanding of the mechanisms involved in symptom development associated with Lso infection and their timing. These insights can be used to inform breeding strategies aimed at improving crop health, resilience, and productivity.

The ubiquitinome comparative analysis between infected and uninfected samples revealed that infection by LsoB led to increased protein ubiquitination, consistent with our previous findings [12]. Significant changes in proteins associated with ubiquitination and the proteasome were prevalent in response to both haplotypes. Several GO terms related to ubiquitination were enriched in both the LsoA vs Lso-free and LsoB vs Lso-free comparisons. These included polyubiquitin modification-dependent protein binding, ubiquitin binding, proteasome-mediated ubiquitin-dependent protein catabolic process, proteasome core complex, and alpha-subunit complex. There are more than 1,600 genes involved in UPS-related functions in the *Arabidopsis* genome; over 87.5% of these genes encode putative E3 ubiquitin ligases [27]. This diversification of E3 ligases is essential to ensure substrate specificity and plant adaptation to different environmental conditions. Two E3 proteins (A0A3Q7HIV4 HECT-type E3 ubiquitin transferase and A0A3Q7FND9 RING-type E3 ubiquitin transferase) were underrepresented in our study in both infected treatments compared to the control. Furthermore, two additional proteins (A0A3Q7G8M7 RING-type E3 ubiquitin transferase and A0A494G9Z5 RING-type E3 ubiquitin transferase) were also underrepresented in the LsoB-infected treatment compared to the control. Four other proteins annotated as containing RING-finger domains (K4BDE8 RING-finger protein; A0A3Q7GJU4 RING-type domain-containing protein; A0A3Q7HW09 RING-type domain-containing protein; and A0A3Q7FR15 RING-type domain-containing protein) were also underrepresented, and one (A0A3Q7I2E5 RING-type domain-containing protein) was overrepresented in LsoB compared to the control. E3 ligases are critical proteins because they control ubiquitination efficiency and specificity [28]. Changes in E3 ligases can lead to infection and disease development by targeting the degradation of immune-related proteins or resulting in the accumulation of misfolded proteins and cellular dysfunction. The severity of the symptoms associated with LsoB infection could be linked to the overrepresentation of these proteins in the LsoB-infected vs control ubiquitinome comparison. Although Gly–Gly ubiquitination has classically been associated with proteasome‑mediated degradation through K48‑linked polyubiquitin chains, the ubiquitination signatures detected in our dataset could also be associated with non‑proteolytic regulatory functions, including immune signaling, membrane trafficking, and transcriptional control. In this context, Gly–Gly modification likely reflects dynamic remodeling of host signaling networks rather than a simple increase in protein turnover.

Two RAD23 protein accessions (A0A3Q7EYX0 and Q9STA6) were differentially represented in the LsoB vs Lso-free and LsoA vs Lso-free comparisons. These results are significant because these accessions correspond to RAD23 proteins that interact with Lso HPE1 [12]. To assess whether these differences could be associated with differential gene regulation rather than ubiquitination, we evaluated the expression of the four Rad23 genes in tomato plants by RT-qPCR. While the transcript level might not correlate with the protein level, we did not find differences in the expression of these four genes between the Lso treatments. Western blotting following transient expression confirmed that RAD23d was undetectable in LsoA-infected plants but not when plants were infected with LsoB. These results suggest that LsoA and LsoB affect Rad23d accumulation differently. Previously, we identified LsoA and LsoB HPE1 proteins that interact with Rad23c, Rad23d, and Rad23e [12]. Therefore, this difference in Rad23d accumulation might be driven by a different mechanism or other effectors not yet characterized. These results suggest that LsoA and LsoB may manipulate the RAD23-mediated degradation pathway differently.

The accumulation of PLANT UBX DOMAIN-CONTAINING PROTEIN 4 and HOP-INTERACTING PROTEIN THI111 proteins was also evaluated; both proteins were undetectable in LsoB-infected plants. UBX proteins are cofactors of members of the AAA ATPase p97 family, which are involved in extracting proteins from the endoplasmic reticulum (ER) during the endoplasmic reticulum-associated protein degradation (ERAD) process [29]. HOP-INTERACTING PROTEIN THI111 contains domains that are characteristic of DDI1 proteins, which, similar to RAD23 proteins, shuttle ubiquitinated proteins to the 26S proteasome for degradation [30]. Therefore, degradation of PLANT UBX DOMAIN-CONTAINING PROTEIN 4 and HOP-INTERACTING PROTEIN THI111 in LsoB-infected plants could be linked to the accumulation of ubiquitinated proteins in those plants. While the biological causes and implications of these differences remain unknown, these regulations contribute to the differences in symptoms associated with each haplotype. Our results highlight the important role of ubiquitination and the UPS in the plant response to Lso and underscore the potential regulation of multiple ubiquitination and degradation pathways.

Overall, the results obtained by Western blot analysis showed that Lso infection affected the degradation of key proteasome-related proteins. Furthermore, the results revealed that infection by LsoA and LsoB can lead to different degradation patterns. These results do not directly confirm the ubiquitinome analyses because ubiquitination does not always lead to degradation; indeed, many non-proteolytical functions of ubiquitination have been uncovered [31]. The expression analyses were performed in *N. benthamiana*, a host susceptible to Lso infection. While LsoB causes more severe symptoms in tomato and *N. benthamiana*, differences in plant responses to Lso infection could be expected between these two hosts*.* Observing similar expression patterns among key ubiquitination-related proteins in a different host confirms the biological significance of the ubiquitinome dataset. Further investigation into the specific roles of these proteins in ubiquitin-dependent pathways could provide deeper insights into their contributions to infection and disease progression.

Several proteins were differentially represented in LsoA- and LsoB-infected samples. For example, superoxide dismutase, a protein involved in controlling oxidative stress in plants [32], was over-represented in LsoB compared to Lso-free but under-represented in LsoA compared to Lso-free. ROS are key signaling and defense molecules involved in the plant response to pathogens, and their accumulation has been linked to symptom development upon CLas or Lso infection. For instance, higher oxidative stress correlates with increased severity of CLas-associated symptoms [33, 34], while four weeks after tomato infection, there are differences in ROS accumulation between LsoA- and LsoB-infected plants [11]. Several other proteins associated with oxidative stress were also identified as enriched during LsoB infection. These enzymes might be linked to the previously mentioned differential accumulation of ROS molecules in LsoA- and LsoB-infected plants. Additionally, Liberibacter infection has been linked to water deficit [35, 36], which is also associated with oxidative stress [37]; therefore, it is not surprising to find enriched terms related to water stress in LsoB-infected plants.

Among the differentially represented proteins, patellin-3 was overrepresented in LsoB compared to Lso-free but underrepresented in LsoA compared to Lso-free. Patellins are multifunctional proteins involved in development, response to stress, and membrane trafficking. Some patellin proteins were shown to interact with the alfalfa mosaic virus movement protein and could impact virus cell-to-cell movement [38, 39]. Lso is a phloem-restricted bacterium with a distribution pattern in plants similar to that of phloem-restricted viruses [40]. However, after the fourth week of infection, the LsoA and LsoB distributions are different in tomato plants [7]. Lso movement needs to be studied in more detail, and patellin proteins are promising candidates to play a role in Lso distribution in plants.

In conclusion, our study cataloged the tomato leaf ubiquitinome in response to Lso infection, providing a snapshot of protein ubiquitination and abundance across three conditions. This study expands the inventory of tomato proteins that can be ubiquitinated and of the ubiquitinated sites within these proteins. Ubiquitination is involved in protein turnover; therefore, part of the observed changes may reflect alterations in overall protein abundance rather than true differences in ubiquitination levels. Additionally, the ubiquitinated proteome includes newly synthesized proteins that failed the "quality check"; therefore, some of the changes identified here might be linked to the regulatory role of ubiquitination, while others might be linked to ER stress. Nonetheless, the differentially represented ubiquitinated proteins are likely associated with the differences in disease progression and immune responses elicited by the two Lso haplotypes. Liberibacter, like other pathogens, can affect protein synthesis and cause ER stress.

## Materials and Methods

### Plant Material

Tomato, *Solanum lycopersicum* L. ‘Moneymaker’ (Thompson & Morgan Inc., Jackson, NJ) and *Nicotiana benthamiana* plants were grown from seeds in plastic pots containing Jolly Gardener ProLine C/25 soil. The plants were fertilized with Miracle-Gro Water-Soluble Tomato Plant Food at the label rate (18—18—21 NPK; Scotts Miracle-Gro Company, Marysville, OH). Throughout the experiment, the plants were maintained on light shelves at room temperature (21—24 °C) and under a 16-h light:8-h dark photoperiod. They were watered twice a week.

### Lso infection and detection in tomato and Nicotiana benthamiana plants

Five-week-old tomato plants were infected by caging four adult psyllids from the LsoA- or LsoB-infected colonies on a lower-tier leaf using mesh jewelry bags. Control plants were infested with four Lso-free psyllids. The psyllids were removed after seven days. There were four plants per infestation treatment. Lso-Free, LsoA, and LsoB colonies were tested regularly by evaluating individual insects from the colony by PCR as described by Yao, et al. [41]. Lso-free were always 100% Lso-free, while insects from the infected colonies commonly displayed 100% infection.

Four weeks after the removal of the psyllids, a top-tier leaf was cut and flash-frozen in liquid nitrogen for the ubiquitinome. This time point was chosen because it is before major symptoms develop [7], but transcriptomic reprogramming has already occurred [19], i.e., the plant is responding to the infection. Earlier time points might be marked by the responses of the plant to psyllid feeding [42]. Beyond this time point, Lso-infected plants develop increasingly severe symptoms, and by 6 weeks post-infection, LsoB-infected plants exhibit a marked reduction in the number of surviving leaves and overall poor plant condition, with plant death often occurring within the following two weeks [7].

Plant tissue was also collected to confirm Lso infection. DNA was extracted as described in Levy, et al. [40] and used as a PCR template to verify the presence of Lso infection using the LsoF/OI2 and SSR1 primers [43, 44]. A similar method was used to generate infected *Nicotiana benthamiana* plants, using 10 nymphs per plant.

### Differential ubiquitination analysis by LC–MS/MS

Two hundred fifty milligrams of tissue was collected, flash-frozen in liquid nitrogen, and shipped to the Duke Proteomics and Metabolomics Core Facility for sample preparation, data collection, and data analysis.

Briefly, plant samples were homogenized by bead beating in 8 M urea and 50 mM ammonium bicarbonate. After debris removal and the Bradford assay, the normalized samples were reduced with 10 mM DTT and alkylated with 20 mM iodoacetamide. The proteins were digested overnight at 37 °C using 5 µg/µL sequencing-grade trypsin (Promega). The samples were then acidified with TFA and subjected to C18 clean-up (Sep-Pak, 50 mg bed, Waters Corporation, Milford, MA). Then, all samples were lyophilized to dryness. The ubiquitinated peptides were enriched using the PTMScan Ubiquitin Remnant Motif Kit (Cell Signaling Technology) following the manufacturer’s protocol.

Quantitative LC–MS/MS was performed using an EvoSep One UPLC system coupled to an Orbitrap Astral high-resolution accurate mass tandem mass spectrometer (Thermo Fisher Scientific). Briefly, each sample loaded with EvoTip was eluted onto a 1.5 µm EvoSep 150 µm ID × 15 cm performance column (EvoSep, Denmark) using the SPD30 gradient at 55 °C. Data collection on the mass spectrometer was performed in data-independent acquisition (DIA) mode with a r = 240,000 (@ m/z 200) full MS scan from m/z 380—980 with a target automatic gain control (AGC) value of 4e^5^ ions. Fixed DIA windows of 4 m/z from m/z 380 to 980 DIA MS/MS scans were acquired with a target AGC value of 5e^4^ and a max fill time of 6 ms. For all MS2 scans, an HCD collision energy setting of 27% was used. The total analysis cycle time for each sample injection was approximately 44 min. For each proteomic analysis, SPQC pools created from an equal portion of individual samples were run at the beginning, middle, and end of the study.

Data were imported into Spectronaut (Biognosys, Switzerland), and individual LC–MS data files were aligned based on the accurate mass and retention time of detected precursor and fragment ions. Relative peptide abundance was measured based on MS2 fragment ions of selected ion chromatograms of the aligned features across all runs. The MS/MS data were searched against a TrEMBL *S. lycopersicum* database (downloaded in 2024), a common contaminant/spiked protein database (bovine albumin, bovine casein, yeast ADH, etc.), and an equal number of reversed sequence “decoys” for false discovery rate determination. The directDIA algorithm was used to create a library within Spectronaut, and then Pulsar was used to perform database searches. Database search parameters included fixed modification on Cys (carbamidomethyl) with variable modification on Met (oxidation) and lysine (GG). Full trypsin enzyme rules were used along with 10 ppm mass tolerances on precursor ions and 20 ppm on product ions. Spectral annotation was set at a maximum 1% peptide false discovery rate based on q-value calculations. Razor rules were applied for peptide homology, where a peptide matching multiple different proteins was exclusively assigned to the protein that had the most identified peptides. Protein homology was addressed by grouping proteins that had the same set of peptides to account for their identification. A master protein within a group was assigned based on percentage coverage. Raw intensity values for each precursor were tabulated from Spectronaut detection software, which has its own criteria for detected peaks; no value (i.e., a blank cell) was assigned if the criteria were not met. For missing values, the following imputation strategy was used. First, peptides with the sequence but different precursor charge states were summed to give a single peptide value. If less than half of the values were missing in a biological group, values were imputed with an intensity derived from a normal distribution of all values defined by measured values within the same intensity range (20 bins). If greater than half of the values were missing for a peptide in a group and the peptide intensity was > 5e^6^, then it was concluded that the peptide was misaligned, and its measured intensity was set to 0. All remaining missing values are imputed with the lowest 2% of all detected values. Prior to normalization, a filter was applied such that a peptide was removed if it was not measured at least twice across all samples and in at least 75% of the replicates in any one single group (3 samples). After that filter, samples were total intensity normalized (total intensity of all peptides for a sample were summed, then normalized across all samples), excluding trypsin, alcohol dehydrogenase, and keratin. These data were then filtered to remove all non-GlyGly peptides, the remaining data were subjected to trimmed-mean normalization in which the top and bottom 10% of the signals were excluded, and the average of the remaining values was used to normalize across all samples. The normalized GlyGLy peptides were used for the analysis. Fold-changes between the various groups based on GlyGly peptide expression values and a two-tailed heteroscedastic t-test on log2-transformed data were calculated. A corrected p-value < 0.05 was used to identify differential peptides.

### Enrichment analysis

Gene Ontology (GO) and Kyoto Encyclopedia of Genes and Genomes (KEGG) pathway enrichment analyses among the differential proteins were conducted using DAVID [45, 46]. Terms were considered enriched if the false discovery rate (FDR) was lower than 0.05. The GO and KEGG analyses were performed using the Direct database.

### Data visualization

A Venn diagram was constructed using Venny 2.1 [47] and modified using Microsoft 365 Copilot. It included all over- and under-represented accessions in the Lso-free vs LsoA, Lso-free vs LsoB, and LsoA vs LsoB comparisons. Enriched GO terms were visualized using RStudio (version 2024.09.1 + 394) with the ggplot2 and ggrepel packages [48, 49]. KEGG pathways [50] were modified by adding color stars representing different comparisons.

### Agroinfiltration-mediated transient expression in Nicotiana benthamiana leaves

To validate the differential expression of selected proteins, we cloned the following genes for overexpression: PLANT UBX DOMAIN-CONTAINING PROTEIN 4 (Solyc07g049260.2, A0A3Q7HDF7), HOP-INTERACTING PROTEIN THI111 (Solyc10g005890.3.1, G8Z278), NEDD8-CONJUGATING ENZYME UBC12 (Solyc07g066080.2, Q9FT39), RAD23a (Solyc03g117780.2, A0A3Q7GIA3), RAD23c (Solyc04g007120.2, A0A3Q7FVH0), RAD23d (Solyc02g063130.2, A0A3Q7EYX0), and RAD23e (Solyc02g085840.2, Q9STA6). Each gene was individually subcloned and inserted into the pMDC32-HA vector using the in-fusion method (Vazyme, Irvine, CA) (Table S7).

The resulting constructs were separately introduced into *Agrobacterium tumefaciens* strain GV3101. Each gene was transiently expressed in Lso-free, LsoA-, or LsoB-infected *N. benthamiana* leaves via agroinfiltration. Briefly, cultured *A. tumefaciens* cells were resuspended in infiltration buffer (10 mM MES, pH 5.6, 10 mM MgCl₂, and 100 μM acetosyringone) to a final OD₆₀₀ of 0.8. After incubation at 25 °C for 3 h in the dark, the suspensions were infiltrated into the leaves using a 1 mL syringe. At 60 h post-infiltration, the leaves were infiltrated with 50 μM MG132 dissolved in DMSO or with DMSO as the control. Twelve hours later, leaf samples were collected. Total proteins were extracted using 50 mM Tris–HCl (pH 7.5), 150 mM NaCl, 1% Triton X-100, 1 mM DTT, 1 mM PMSF, and a complete protease inhibitor cocktail (Roche). Western blot analyses were conducted using anti-HA (Invitrogen, Carlsbad, CA, USA) and HRP-conjugated goat anti-rabbit IgG (H&L) secondary (Invitrogen) antibodies. The blots were stained with Ponceau S to verify equal loading.

### RNA extraction, cDNA preparation, and qPCR

Expression of the Rad23 genes was quantified from uninfested and Lso-Free, LsoA- and LsoB-infested tomato plants. Samples from the top leaves were collected 4 weeks after infestation. There were five plants per treatment. RNA was extracted from 100 mg of flash-frozen leaf tissue using an RNeasy Plant Mini kit (Qiagen, Valencia, CA) according to the manufacturer's instructions. The RNA was treated with Turbo DNase (Thermo Fisher Scientific) and used to synthesize complementary DNA (cDNA) with the Verso cDNA Synthesis kit (Thermo Fisher Scientific) and anchored-Oligo (dT) primers according to the manual. cDNA was used as a template for quantitative PCR (qPCR) analyses using gene-specific primers and the SYBR green supermix kit (Bioline, Taunton, MA). The reaction mixture (10 μL) contained 5 μL of Power SYBR Green PCR Master Mix, 2 μL of cDNA template, 0.5 μL of forward and reverse primers (250 nM each, Table S7), and 2 μL of deionized distilled water. The RT-qPCR program was 95 °C for 2 min, followed by 40 cycles at 95 °C for 5 s and 60 °C for 30 s. RT-qPCR assays were performed using a QuantStudio 6 Flex Real-Time PCR System (Applied Biosystems). Elongation factor 1 was used as a reference gene [51]. All reactions were performed in triplicate. The expression of the candidate genes was calculated using the ∆Ct method. The results were analyzed with RStudio (version 4.4.2) with one-way ANOVA.

## Supplementary Information

Supplementary Material 1. Figure S1: Diagram showing GO term and KEGG enrichment analyses.

Supplementary Material 2. Figure S2: RT-qPCR of Rad23 genes in tomato plants 4 weeks after infestation with Lso-Free, LsoA- or LsoB-infected psyllids. The expression of Rad23a, Rad23c, Rad23d, and Rad23e was evaluated in five plants per treatment using Ef1α as the reference gene. For ∆∆Ct calculations, uninfected control plants were used as the reference condition.

Supplementary Material 3. Table S1: Ubiquitinome comparison between LsoA and Lso-free.

Supplementary Material 4. Table S2: Ubiquitinome comparison between LsoB and Lso-free.

Supplementary Material 5. Table S3: Ubiquitinome comparison between LsoA and LsoB.

Supplementary Material 6. Table S4: Common accessions between the LsoA vs Lso-free and LsoB vs Lso-free comparisons.

Supplementary Material 7. Table S5: Enriched GO terms and KEGG pathways among the differentially represented proteins in the LsoA vs Lso-free and LsoB and Lso-free comparisons.

Supplementary Material 8. Table S6: Number of up- and down-regulated accessions and multiple motifs.

Supplementary Material 9. Table S7: The primers used for cloning into the vector pMDC32-HA.

## Data Availability

All available data are presented in the manuscript.

## References

[1] Haapalainen M. Biology and epidemics of *Candidatus Liberibacter* species, psyllid-transmitted plant-pathogenic bacteria. Ann Appl Biol. 2014;165:172–98. 10.1111/aab.12149.

[2] Wang N, Pierson EA, Setubal JC, Xu J, Levy JG, Zhang YZ, et al. The *Candidatus Liberibacter*-host interface: insights into pathogenesis mechanisms and disease control. Annu Rev Phytopathol. 2017;55:451–82. 10.1146/annurev-phyto-080516-035513. 28637377 10.1146/annurev-phyto-080516-035513

[3] Raddadi N, Gonella E, Camerota C, Pizzinat A, Tedeschi R, Crotti E, et al. *Candidatus Liberibacter europaeus* sp. nov. that is associated with and transmitted by the psyllid *Cacopsylla pyri* apparently behaves as an endophyte rather than a pathogen. Environ Microbiol. 2011;13:414–26. 10.1111/j.1462-2920.2010.02347.x. 21040355 10.1111/j.1462-2920.2010.02347.x

[4] Glynn JM, Islam M, Bai Y, Lan S, Wen A, Gudmestad NC, et al. Multilocus sequence typing of *Candidatus Liberibacter solanacearum* isolates from North America and New Zealand. J Plant Pathol. 2012;94:223–8.

[5] Liefting L, Perez-Egusquiza Z, Clover G, Anderson J. A new *Candidatus Liberibacter* species in *Solanum tuberosum* in New Zealand. Plant Dis. 2008;92:1474–1474. 10.1094/pdis-92-10-1474a. 30769561 10.1094/PDIS-92-10-1474A

[6] Lin H, Islam MS, Bai Y, Wen A, Lan S, Gudmestad NC, et al. Genetic diversity of *Candidatus Liberibacter solanacearum* strains in the United States and Mexico revealed by simple sequence repeat markers. Eur J Plant Pathol. 2012;132:297–308. 10.1007/s10658-011-9874-3.

[7] Mendoza Herrera A, Levy J, Harrison K, Yao J, Ibanez F, Tamborindeguy C. Infection by *Candidatus Liberibacter solanacearum* haplotypes A and B in *Solanum lycopersicum* ‘Moneymaker.’ Plant Dis. 2018;102:2009–15. 10.1094/PDIS-12-17-1982-RE. 30133358 10.1094/PDIS-12-17-1982-RE

[8] Levy JG, Oh J, Mendoza Herrera MA, Parida A, Lao L, Starkey J, et al. A *Candidatus Liberibacter solanacearum* haplotype B-specific family of candidate bacterial effectors. Phytopathology. 2023;113:1708–15. 10.1094/phyto-11-22-0438-v. 37665323 10.1094/PHYTO-11-22-0438-V

[9] Levy JG, Parida AP, Oh J, Mendoza-Herrera A, Shaw BD, Tamborindeguy C. *Candidatus liberibacter solanacearum* protein CKC_05770 interacts in vivo with tomato APX6 and APX7. Sci Rep. 2025;15:10826. 10.1038/s41598-025-93367-w. 40155471 10.1038/s41598-025-93367-w PMC11953316

[10] Reyes Caldas PA, Zhu J, Breakspear A, Thapa SP, Toruño TY, Perilla-Henao LM, et al. Effectors from a bacterial vector-borne pathogen exhibit diverse subcellular localization, expression profiles, and manipulation of plant defense. Mol Plant-Microbe Interact. 2022;35:1067–80. 10.1094/mpmi-05-22-0114-r. 35952362 10.1094/MPMI-05-22-0114-R PMC9844206

[11] Oh J, Levy JG, Mendoza Herrera A, Tamborindeguy C. HPE21, a liberibacter effector modulating H_2_O_2_ accumulation in tomato plants. Physiol Mol Plant Pathol. 2026;142:103113. 10.1016/j.pmpp.2026.103113.

[12] Kan C-C, Mendoza-Herrera A, Levy J, Hull JJ, Fabrick JA, Tamborindeguy C. HPE1, an effector from zebra chip pathogen interacts with tomato proteins and perturbs ubiquitinated protein accumulation. Int J Mol Sci. 2021;22:9003. 34445707 10.3390/ijms22169003 PMC8396652

[13] Levy JG, Gross R, Mendoza-Herrera A, Tang X, Babilonia K, Shan L, et al. Lso-HPE1, an effector of ‘*Candidatus Liberibacter solanacearum*’, can repress plant immune response. Phytopathology. 2020;110:648–55. 31697198 10.1094/PHYTO-07-19-0252-R

[14] Langin G, González-Fuente M, Üstün S. The plant ubiquitin–proteasome system as a target for microbial manipulation. Annu Rev Phytopathol. 2023;61:351–75. 10.1146/annurev-phyto-021622-110443. 37253695 10.1146/annurev-phyto-021622-110443

[15] Kitazawa Y, Iwabuchi N, Maejima K, Sasano M, Matsumoto O, Koinuma H, et al. A phytoplasma effector acts as a ubiquitin-like mediator between floral MADS-box proteins and proteasome shuttle proteins. Plant Cell. 2022;34:1709–23. 10.1093/plcell/koac062. 35234248 10.1093/plcell/koac062 PMC9048881

[16] MacLean AM, Orlovskis Z, Kowitwanich K, Zdziarska AM, Angenent GC, Immink RGH, et al. Phytoplasma effector SAP54 hijacks plant reproduction by degrading MADS-box proteins and promotes insect colonization in a RAD23-dependent manner. PLOS Biol. 2014;12:e1001835. 10.1371/journal.pbio.1001835. 24714165 10.1371/journal.pbio.1001835 PMC3979655

[17] Rape M. Ubiquitylation at the crossroads of development and disease. Nat Rev Mol Cell Biol. 2018;19:59–70. 10.1038/nrm.2017.83. 28928488 10.1038/nrm.2017.83

[18] Beck DB, Wu Z, Patel BA, Ferrada MA, Sikora KA, Ombrello A, et al. Somatic mutations in a single residue of UBA1 cause VEXAS, a severe adult-onset rheumatic disease associated with myeloid dysplasia. Blood. 2020;136:36–7.32430502

[19] Harrison K, Levy JG, Tamborindeguy C. Effects of '*Candidatus Liberibacter solanacearum*’ haplotypes A and B on tomato gene expression and geotropism. BMC Plant Biol. 2022;22:156. 10.1186/s12870-022-03505-z. 35354405 10.1186/s12870-022-03505-z PMC8966271

[20] Spoel SH, Dong X. How do plants achieve immunity? Defence without specialized immune cells. Nat Rev Immunol. 2012;12:89–100. 10.1038/nri3141. 22273771 10.1038/nri3141

[21] Zhou J-M, Zhang Y. Plant immunity: danger perception and signaling. Cell. 2020;181:978–89. 32442407 10.1016/j.cell.2020.04.028

[22] Zhou B, Zeng L. Conventional and unconventional ubiquitination in plant immunity. Mol Plant Pathol. 2017;18:1313–30. 27925369 10.1111/mpp.12521 PMC6638253

[23] Cheng YT, Li X. Ubiquitination in NB-LRR-mediated immunity. Curr Opin Plant Biol. 2012;15:392–9. 22503756 10.1016/j.pbi.2012.03.014

[24] Adams EH, Spoel SH. The Ubiquitin–Proteasome System as a transcriptional regulator of plant immunity. J Exp Bot. 2018;69:4529–37. 29873762 10.1093/jxb/ery216

[25] Carpenter MA, Frew TJ, Boldingh HL, Nardozza S, Shaw ML, Thomson SJ, et al. Transcriptomic and metabolomic profiling of the potato plant response to Zebra Chip Disease. PLoS ONE. 2025;20:e0328035. 10.1371/journal.pone.0328035. 40632792 10.1371/journal.pone.0328035 PMC12240308

[26] Levy JG, Mendoza A, Miller JC, Tamborindeguy C, Pierson EA. Global gene expression in two potato cultivars in response to ‘Candidatus *Liberibacter solanacearum*’ infection. BMC Genomics. 2017;18:960. 10.1186/s12864-017-4313-2. 29228896 10.1186/s12864-017-4313-2 PMC5725879

[27] Craig A, Ewan R, Mesmar J, Gudipati V, Sadanandom A. E3 ubiquitin ligases and plant innate immunity. J Exp Bot. 2009;60:1123–32. 19276192 10.1093/jxb/erp059

[28] Zheng N, Shabek N. Ubiquitin ligases: structure, function, and regulation. Annu Rev Biochem. 2017;86:129–57. 10.1146/annurev-biochem-060815-014922. 28375744 10.1146/annurev-biochem-060815-014922

[29] Schuberth C, Buchberger A. UBX domain proteins: major regulators of the AAA ATPase Cdc48/p97. Cell Mol Life Sci. 2008;65:2360–71. 10.1007/s00018-008-8072-8. 18438607 10.1007/s00018-008-8072-8 PMC11131665

[30] Fabijan A, Polis B, Zawadzka-Fabijan A, Korabiewska I, Zakrzewski K, Nowosławska E, et al. Domains in action: understanding Ddi1’s diverse functions in the Ubiquitin-Proteasome System. Int J Mol Sci. 2024;25:4080. 38612889 10.3390/ijms25074080 PMC11012796

[31] Liao Y, Sumara I, Pangou E. Non-proteolytic ubiquitylation in cellular signaling and human disease. Commun Biol. 2022;5:114. 10.1038/s42003-022-03060-1. 35136173 10.1038/s42003-022-03060-1 PMC8826416

[32] Alscher RG, Erturk N, Heath LS. Role of superoxide dismutases (SODs) in controlling oxidative stress in plants. J Exp Bot. 2002;53:1331–41. 11997379

[33] Tang L, Vashisth T. New insight in Huanglongbing-associated mature fruit drop in citrus and its link to oxidative stress. Sci Hortic. 2020;265:109246.

[34] Pandey SS, Li J, Oswalt C, Wang N. Dynamics of ‘*Candidatus Liberibacter asiaticus*’ growth, concentrations of reactive oxygen species, and ion leakage in huanglongbing-positive sweet orange. Phytopathology. 2024;114:961–70. 10.1094/phyto-08-23-0294-kc. 38478730 10.1094/PHYTO-08-23-0294-KC

[35] da Silva JR, Boaretto RM, Lavorenti JAL, Dos Santos BCF, Coletta-Filho HD, Mattos D Jr. Effects of deficit irrigation and Huanglongbing on sweet orange trees. Front Plant Sci. 2021;12:731314. 10.3389/fpls.2021.731314. 34721459 10.3389/fpls.2021.731314 PMC8554030

[36] Cohen A, Basu S, Crowder DW. Drought stress affects interactions between potato plants, psyllid vectors, and a bacterial pathogen. FEMS Microbiol Ecol. 2023;99:fiac142. 10.1093/femsec/fiac142. 10.1093/femsec/fiac142 36416808

[37] Kar RK. Plant responses to water stress: role of reactive oxygen species. Plant Signal Behav. 2011;6:1741–5. 10.4161/psb.6.11.17729. 22057331 10.4161/psb.6.11.17729 PMC3329347

[38] Peiro A, Izquierdo-Garcia AC, Sanchez-Navarro JA, Pallas V, Mulet JM, Aparicio F. Patellins 3 and 6, two members of the Plant Patellin family, interact with the movement protein of Alfalfa mosaic virus and interfere with viral movement. Mol Plant Pathol. 2014;15:881–91. 10.1111/mpp.12146. 24751128 10.1111/mpp.12146 PMC6638666

[39] Zhou H, Duan H, Liu Y, Sun X, Zhao J, Lin H. Patellin protein family functions in plant development and stress response. J Plant Physiol. 2019;234–235:94–7. 10.1016/j.jplph.2019.01.012. 30690193 10.1016/j.jplph.2019.01.012

[40] Levy J, Ravindran A, Gross D, Tamborindeguy C, Pierson E. Translocation of ‘*Candidatus Liberibacter solanacearum*’, the zebra chip pathogen, in potato and tomato. Phytopathology. 2011;101:1285–91. 21770778 10.1094/PHYTO-04-11-0121

[41] Yao J, Saenkham P, Levy J, Ibanez F, Noroy C, Mendoza A, et al. Interactions ‘*Candidatus Liberibacter solanacearum*’ – *Bactericera cockerelli*: haplotype effect on vector fitness and gene expression analyses. Front Cell Infect Microbiol. 2016;6:62. 27376032 10.3389/fcimb.2016.00062 PMC4899927

[42] Harrison K, Mendoza-Herrera A, Levy JG, Tamborindeguy C. Lasting consequences of psyllid (*Bactericera cockerelli* L.) infestation on tomato defense, gene expression, and growth. BMC Plant Biol. 2021;21:114. 33627099 10.1186/s12870-021-02876-z PMC7905647

[43] Li W, Abad JA, French-Monar RD, Rascoe J, Wen A, Gudmestad NC, et al. Multiplex real-time PCR for detection, identification and quantification of '*Candidatus Liberibacter solanacearum*’ in potato plants with zebra chip. J Microbiol Methods. 2009;78:59–65. 10.1016/j.mimet.2009.04.009. 19409423 10.1016/j.mimet.2009.04.009

[44] Li W, Hartung JS, Levy L. Quantitative real-time PCR for detection and identification of *Candidatus Liberibacter* species associated with citrus Huanglongbing. J Microbiol Methods. 2006;66:104–15. 16414133 10.1016/j.mimet.2005.10.018

[45] Huang daW, Sherman BT, Lempicki RA. Systematic and integrative analysis of large gene lists using DAVID bioinformatics resources. Nat Protoc. 2009;4:44–57. 10.1038/nprot.2008.211. 19131956 10.1038/nprot.2008.211

[46] Huang daW, Sherman BT, Lempicki RA. Bioinformatics enrichment tools: paths toward the comprehensive functional analysis of large gene lists. Nucleic Acids Res. 2009;37:1–13. 10.1093/nar/gkn923. 19033363 10.1093/nar/gkn923 PMC2615629

[47] Oliveros JC. Venny. An interactive tool for comparing lists with Venn's diagrams, 2007–2015. https://bioinfogp.cnb.csic.es/tools/venny/index.html

[48] Slowikowski K, Schep A, Hughes S, Lukauskas S, Irisson J-O, Kamvar ZN, Ryan T, Christophe D, Hiroaki Y, Gramme P (2018) Package ggrepel. Automatically position non-overlapping text labels with ‘ggplot2

[49] Wickham H. Data Analysis. In: Wickham H, editor. ggplot2: Elegant Graphics for Data Analysis. Springer; 2016. p. 189–201.

[50] Kanehisa M, Furumichi M, Sato Y, Kawashima M, Ishiguro-Watanabe M. KEGG for taxonomy-based analysis of pathways and genomes. Nucleic Acids Res. 2023;51:D587-d592. 10.1093/nar/gkac963. 36300620 10.1093/nar/gkac963 PMC9825424

[51] Expósito-Rodríguez M, Borges AA, Borges-Pérez A, Pérez JA. Selection of internal control genes for quantitative real-time RT-PCR studies during tomato development process. BMC Plant Biol. 2008;8:131. 10.1186/1471-2229-8-131. 19102748 10.1186/1471-2229-8-131 PMC2629474

