# Supplementary figures and images for: Tomato ubiquitinome in response to ‘Candidatus Liberibacter solanacearum’ haplotypes A and B
Source: Crop Health. 2026 May 11;4(1):15. doi: 10.1007/s44297-026-00075-6 (PMC13161422; doi:10.1007/s44297-026-00075-6)

## LsoA vs Lso-free

## LsoB vs Lso-free

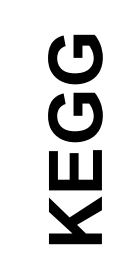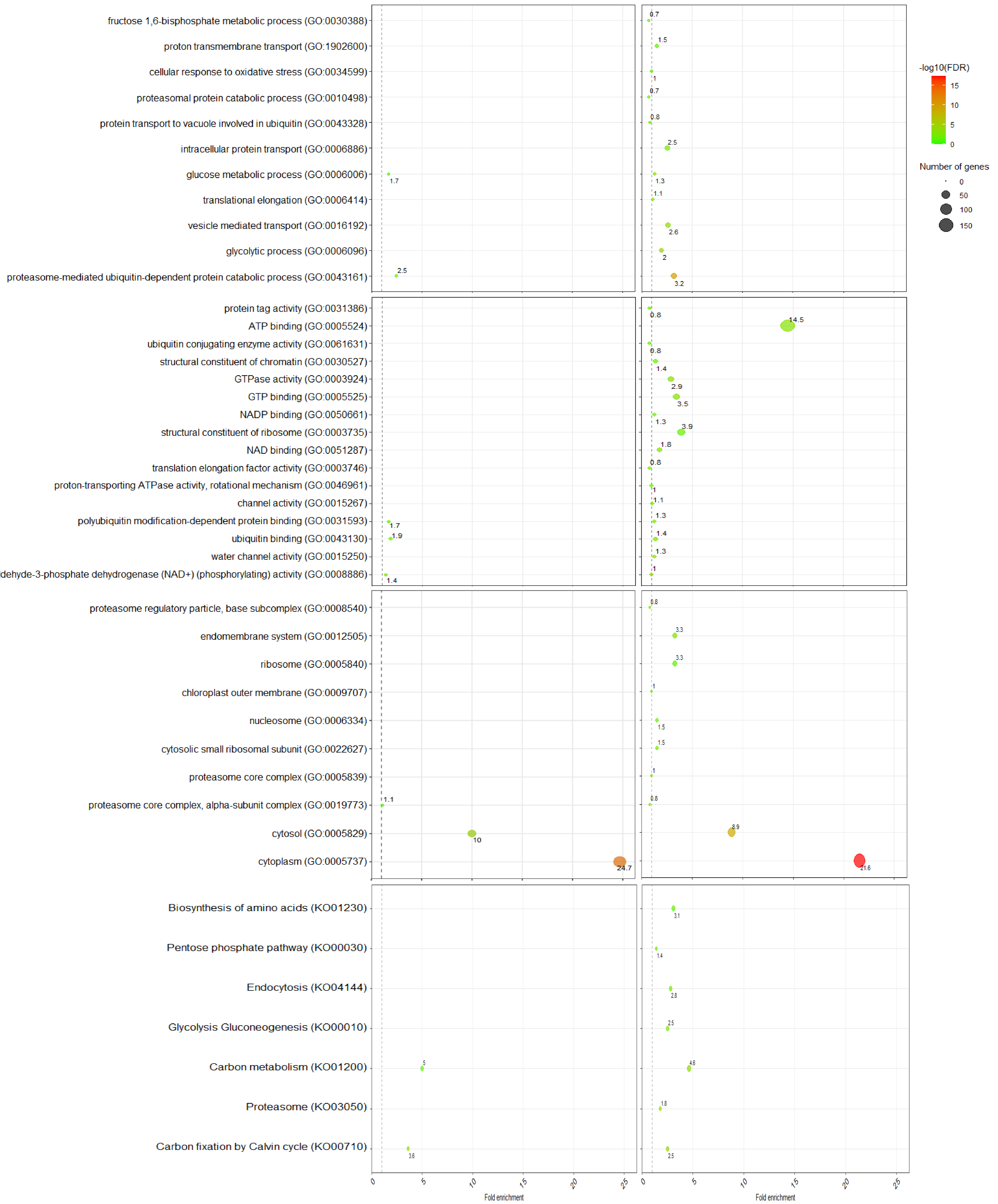

Supplement: Supplementary file 1 — Supplementary Material 1. Figure S1: Diagram showing GO term and KEGG enrichment analyses. [file 44297_2026_75_MOESM1_ESM.pdf]

**
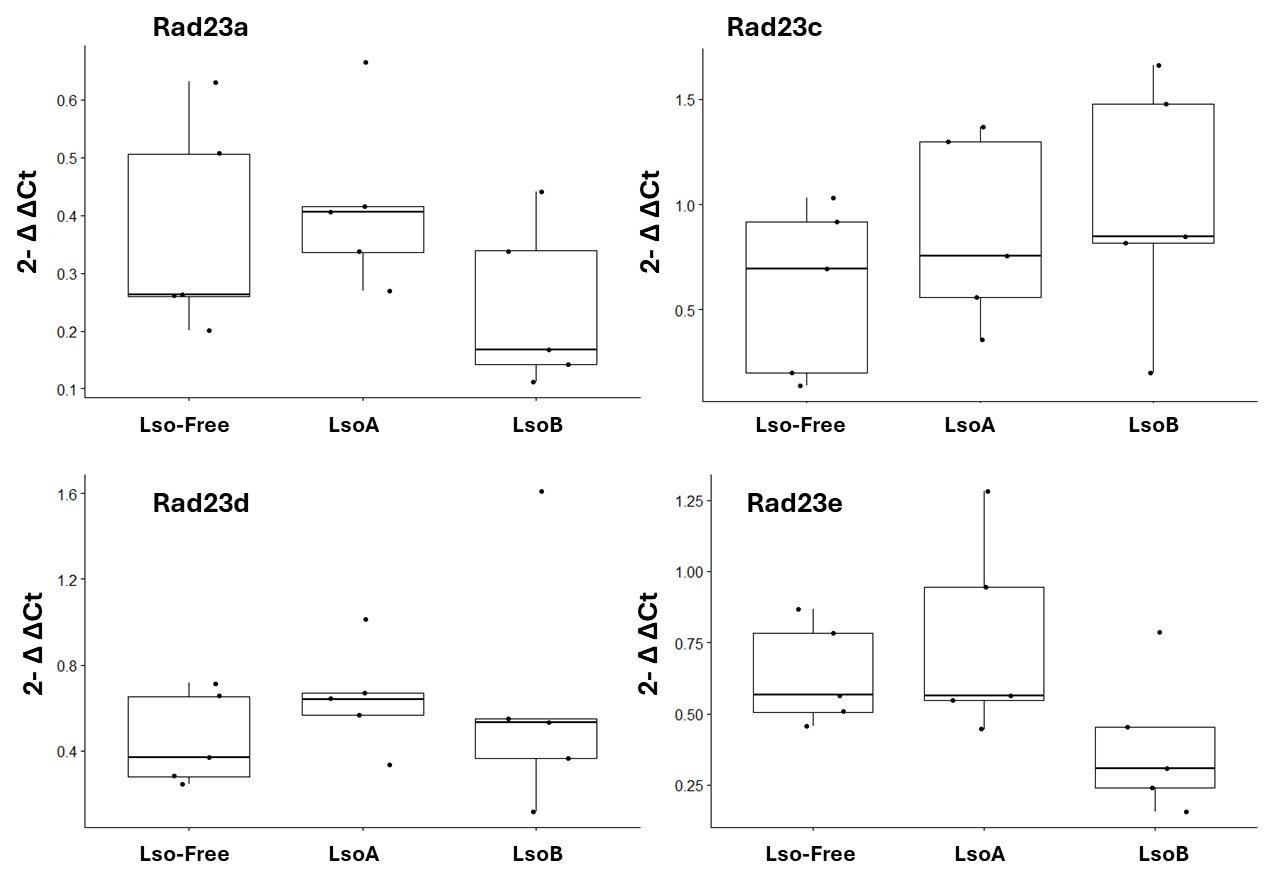
**

Supplement: Supplementary file 2 — Supplementary Material 2. Figure S2: RT-qPCR of Rad23 genes in tomato plants 4 weeks after infestation with Lso-Free, LsoA- or LsoB-infected psyllids. The expression of Rad23a, Rad23c, Rad23d, and Rad23e was evaluated in five plants per treatment using Ef1α as the reference gene. For ∆∆Ct calculations, uninfected control plants were used as the reference condition. [file 44297_2026_75_MOESM2_ESM.docx]
