# Supplementary material for: Tomato ubiquitinome in response to ‘Candidatus Liberibacter solanacearum’ haplotypes A and B
Source: Crop Health. 2026 May 11;4(1):15. doi: 10.1007/s44297-026-00075-6 (PMC13161422; doi:10.1007/s44297-026-00075-6)
Supplement: Supplementary file 8 — Supplementary Material 8. Table S6: Number of up- and down-regulated accessions and multiple motifs. [file 44297_2026_75_MOESM8_ESM.docx]

| \|  \| LsoB Vs LsoFree \| LsoA Vs LsoFree \| LsoB Vs LsoA \| \| --- \| --- \| --- \| --- \| \| Total accession \| **710** \| 357 \| 144 \| \|  \|  \|  \|  \| \| Total down \| 354 (over represented in Lso-Free ) \| 180 (over represented in Lso-Free) \| 61 (over represented in LsoA) \| \| Multiple down \| 78 \| 36 \| 11 \| \| Opposite with majority of down \| 14 \| 1 \| 0 \| \|  \|  \|  \|  \| \| Total up \| 336 (over represented in LsoB) \| 170 (over represented in LsoA) \| 83 (More in LsoB) \| \| Multiple up \| 85 \| 32 \| 14 \| \| Opposite with majority of up \| 10 \| 2 \| 0 \| \|  \|  \|  \|  \| \| Opposite up and down \| 20 \| 7 \| 0 \| \| **M>0.75** \|  \|  \|  \| \| **P<0.05** \|  \|  \|  \| |
| --- | --- | --- | --- | --- | --- | --- | --- | --- | --- | --- | --- | --- | --- | --- | --- | --- | --- | --- | --- | --- | --- | --- | --- | --- | --- | --- | --- | --- | --- | --- | --- | --- | --- | --- | --- | --- | --- | --- | --- | --- | --- | --- | --- | --- | --- | --- | --- | --- | --- | --- | --- | --- | --- | --- | --- | --- |
