# Supplementary material for: Tomato ubiquitinome in response to ‘Candidatus Liberibacter solanacearum’ haplotypes A and B
Source: Crop Health. 2026 May 11;4(1):15. doi: 10.1007/s44297-026-00075-6 (PMC13161422; doi:10.1007/s44297-026-00075-6)
Supplement: Supplementary file 9 — Supplementary Material 9. Table S7: The primers used for cloning into the vector pMDC32-HA. [file 44297_2026_75_MOESM9_ESM.docx]

**Primers used for cloning and qPCR analysis.**

| **Primer Names** | **Sequences 5’-3’** |
| --- | --- |
|  |  |
| DC32-RAD23e-HA-F | AGGACCTCGACTCTAGAGGATCCATGAAGATTTTCGTGAAGACTT |
| DC32-RAD23e-HA-R | GGAACGTCGTATGGGTAAGGCCTCTCGTCAAACTCATGCAAGT |
| DC32-RAD23c-HA-F | AGGACCTCGACTCTAGAGGATCCATGAAGATTTTTGTGAAAACT |
| DC32-RAD23c-HA-R | GGAACGTCGTATGGGTAAGGCCTCTCTTCAAACTCGTGAATGTG |
| DC32-RAD23d-HA-F | AGGACCTCGACTCTAGAGGATCCATGAAGATTTTTGTGAAGACT |
| DC32-RAD23d-HA-R | GGAACGTCGTATGGGTAAGGCCTTTCATCAAACTCATGCATGT |
| DC32-RAD23a-HA-F | AGGACCTCGACTCTAGAGGATCCATGAAGCTCACTGTAAAGAC |
| DC32-RAD23a-HA-R | GGAACGTCGTATGGGTAAGGCCTATCTTCATAATCTCCTGCATG |
| DC32-RAD23e-HA-F | AGGACCTCGACTCTAGAGGATCCATGAAGATTTTCGTGAAGACTT |
| DC32-RAD23e-HA-R | GGAACGTCGTATGGGTAAGGCCTCTCGTCAAACTCATGCAAGT |
| DC32-TH1111-HA-F | AGGACCTCGACTCTAGAGGATCCATGAAGATCACTGTAATGACG |
| DC32-TH1111-HA-R | GGAACGTCGTATGGGTAAGGCCTACCCCCAAATAGATATCCGG |
| DC32-UBX-HA-F | AGGACCTCGACTCTAGAGGATCCATGGAAAATCAGGCAAATCTC |
| DC32-UBX-HA-R | GGAACGTCGTATGGGTAAGGCCTAAGTTTCTGAATCACAACTG |
| DC32-UBC12-HA-F | AGGACCTCGACTCTAGAGGATCCATGGCTTCAAAGAGGATTCAG |
| DC32-UBC12-HA-R | GGAACGTCGTATGGGTAAGGCCTACCCATTGCGTATTTCTGGGT |
|  |  |
| Ef1α qF | TACTGGTGGTTTTGAAGCTG |
| Ef1α qR | AACTTCCTTCACGATTTCATCATA |
| Rad23a-qF | CTTTGAAATTAGGGTTCAGCCATCT |
| Rad23a-qR | ATCAGCAACTGCTGCCCACA |
| Rad23C-qF | AGAACATAGAAACAGTTCAGGGTTC |
| Rad23C-qR | CGAGTGTTGTCCCATCTTTAAGTACC |
| Rad23D-qF | AAACCCGAAGACACGGTTGCT |
| Rad23D-qR | AGCGGGGTAGACATCTTGACCT |
| Rad23e-qF | GAGATCGAAGTGAAACCGGAAGATAG |
| Rad23e-qR | TAGACATCTTGCCCCTGAACTGA |
